# Characteristics and outcomes of COVID-19 patients presumed to be treated with sotrovimab in NHS hospitals in England

**DOI:** 10.1186/s12879-024-09311-2

**Published:** 2024-04-22

**Authors:** Vishal Patel, Bethany Levick, Stephen Boult, Daniel C. Gibbons, Myriam Drysdale, Emily J. Lloyd, Moushmi Singh, Helen J. Birch

**Affiliations:** 1 GSK House, 980 Great West Road, TW8 9GS Brentford, Middlesex UK; 2 Harvey Walsh, Cheshire, UK

**Keywords:** COVID-19, Sotrovimab, Omicron, Monoclonal antibody

## Abstract

**Background:**

The impact of the constantly evolving severe acute respiratory syndrome coronavirus 2 on the effectiveness of early coronavirus disease 2019 (COVID-19) treatments is unclear. Here, we report characteristics and acute clinical outcomes of patients with COVID-19 treated with a monoclonal antibody (mAb; presumed to be sotrovimab) across six distinct periods covering the emergence and predominance of Omicron subvariants (BA.1, BA.2, and BA.5) in England.

**Methods:**

Retrospective cohort study using data from the Hospital Episode Statistics database from January 1–July 31, 2022. Included patients received a mAb delivered by a National Health Service (NHS) hospital as a day-case, for which the primary diagnosis was COVID-19. Patients were presumed to have received sotrovimab based on NHS data showing that 99.98% of COVID-19-mAb-treated individuals received sotrovimab during the study period. COVID-19-attributable hospitalizations were reported overall and across six distinct periods of Omicron subvariant prevalence. Subgroup analyses were conducted in patients with severe renal disease and active cancer.

**Results:**

Among a total of 10,096 patients, 1.0% (*n* = 96) had a COVID-19-attributable hospitalization, 4.6% (*n* = 465) had a hospital visit due to any cause, and 0.3% (*n* = 27) died due to any cause during the acute period. COVID-19-attributable hospitalization rates were consistent among subgroups, and no significant differences were observed across periods of Omicron subvariant predominance.

**Conclusions:**

Levels of COVID-19-attributable hospitalizations and deaths were low in mAb-treated patients and among subgroups. Similar hospitalization rates were observed whilst Omicron BA.1, BA.2, and BA.5 were predominant, despite reported reductions in in vitro neutralization activity of sotrovimab against BA.2 and BA.5.

## Introduction

Coronavirus disease 2019 (COVID-19), which is caused by infection with the severe acute respiratory syndrome coronavirus 2 (SARS-CoV-2), has been associated with a substantial clinical and economic burden worldwide [1, 2].

Sotrovimab is a dual-action Fc-engineered human monoclonal antibody (mAb) that was developed for the treatment of COVID-19 and targets a conserved epitope in the SARS-CoV-2 spike protein distinct from the angiotensin-converting enzyme-2 receptor binding motif [3]. The phase 2/3 COVID-19 Monoclonal antibody Efficacy Trial-Intent to Care Early (COMET-ICE; NCT04545060) trial assessed the efficacy and safety of sotrovimab administered intravenously in high-risk patients with mild-to-moderate COVID-19 and was conducted during a period of wildtype SARS-CoV-2 predominance. Final results for the primary endpoint showed a 79% (95% confidence interval [CI]: 50–91%, *P* < 0.001) relative risk reduction in all-cause > 24-hour hospitalization or death with sotrovimab treatment compared with placebo [4].

Sotrovimab received approval from the European Medicines Authority [5] and Medicines & Healthcare products Regulatory Agency in December 2021 [6] for the ambulatory treatment of mild-to-moderate COVID-19 in adults and adolescents (≥ 12 years of age) who are at increased risk of developing severe disease. Sotrovimab is administered as a day-case intravenous infusion, where the patient is admitted electively with the intention of not using a hospital bed overnight [6]. In addition to sotrovimab, high-risk patients with COVID-19 in the UK can also be eligible for early treatment with nirmatrelvir/ritonavir, molnupiravir, or remdesivir [7, 8].

In vitro pseudotyped viral assays have assessed the neutralization activity of sotrovimab against Omicron variants, with 2.7-, 16-, and 22.6-fold changes in half-maximal inhibitory concentration vs. wild type reported for BA.1, BA.2, and BA.5, respectively [3, 9]. The clinical implications of reduced in vitro neutralization are unknown, and there is no validated clinical pharmacology model for sotrovimab that can reliably predict clinical efficacy from in vitro neutralization.

Here, we describe the real-world use of sotrovimab, as well as clinical outcomes in a population with assumed treatment with sotrovimab for the management of high-risk patients with COVID-19 in National Health Service (NHS) hospitals across England, at times when Omicron BA.1, BA.2, and BA.5 were predominant.

## Methods

### Study design and data source

This was a retrospective cohort study using data from the Hospital Episode Statistics (HES) database. HES is a data warehouse containing records of hospital diagnoses, procedures, treatments, health care resource utilization, and associated costs for all patients admitted to NHS hospitals in England.

### Identification of presumed sotrovimab administration

HES describes diagnoses and procedures associated with episodes of care without direct reporting of pharmacy data. While we were unable to directly ascertain sotrovimab administration, weekly data for individuals receiving COVID-19 treatments showed that, during the study period, the vast majority of non-hospitalized patients being treated with a mAb were actually treated with sotrovimab (30,234 patients out of a total of 30,241 [99.98%]– as per the report published on January 5, 2023) [10]. As such, episodes identified as day-case admissions that were associated with both a primary diagnosis of COVID-19 (International Statistical Classification of Diseases and Related Health Problems 10th revision [ICD-10] U07.1) [11] and a record of intravenous mAb administration (OPCS-4 × 89.2, per NHS Digital guidance) [12] were deemed to represent sotrovimab administration for the purposes of this study. The ICD-10 U07.2 code, which translates to “COVID-19, virus not identified” as per the World Health Organization IDC-10 2019 guidance, was used to confirm absence of COVID-19 diagnosis [11].

### Population

To be eligible for inclusion, patients had to have a record of mAb administration (OPCS-4 × 89.2) within a spell occurring between January 1 and July 31, 2022 that was identified as a day-case admission and associated with a primary diagnosis of COVID-19 (ICD-10 U07.1) in the HES database. The start date of the earliest qualifying spell was considered a patient’s index date and the spell was considered the index spell. To be included in analyses, patients had to be aged ≥ 12 years as of their index date.

To further ensure we identified mAb administrations that were most likely to be sotrovimab, patients whose index spell had a length of stay greater than 1 day or who had another record associated with a mAb administration in the 28 days prior to index (or following the first event other than where given as part of inpatient care in the study period), were excluded from the study.

The baseline period, during which comorbidities were identified, was defined as the 365 days prior to the index date. The 365-day period was chosen so that long-term conditions requiring infrequent hospital visits were identified, and to minimize the capture of conditions no longer affecting patients. Comorbidities were assessed throughout all patient history, apart from active cancer (which was classified as receiving radiotherapy in the 6 months prior to index and/or chemotherapy in the 12 months prior to index) and stem cell transplants (in the 12 months prior to index) [13]. A patient’s acute period, during which outcomes were evaluated, was defined as the 28 days following the index date.

### Study periods

The study was divided into six distinct periods that reflected the dynamics of Omicron BA.1, BA.2, and BA.5 subvariant activity (Table 1). These periods were defined based on the prevalence of SARS-CoV-2 infections with these variants (ecological study design), informed by sequencing data that were reported in the weekly technical briefing reports published by the UK Health Security Agency [14].

**Table 1:** Study periods

Period	Omicron subvariant prevalence [13]	Duration
Period 1	BA.2 prevalence < 25% (predominant variant: BA.1)	January 1–February 6, 2022
Period 2	BA.2 prevalence ≥ 25% but < 75%	February 7–February 27, 2022
Period 3	BA.2 prevalence ≥ 75%	February 28–May 1, 2022
Period 4^a^	BA.5 prevalence < 25%	May 2–May 31, 2022
Period 5	BA.5 prevalence ≥ 25% but < 75%	June 1–July 3, 2022
Period 6	BA.5 prevalence ≥ 75%	July 4–July 31, 2022

^a^ Starting with period 4, a declining prevalence of Omicron BA.2 and increasing prevalence of Omicron BA.5 was observed; the main circulating variants were Omicron BA.4 and BA.5

### Patient characteristics and study outcomes

Patient characteristics (age, sex, ethnicity, presence of specific comorbidities that indicate a high risk of developing severe COVID-19, and previous admissions for COVID-19) collated from already available baseline data or information captured during the index spell were described for the overall cohort.

The primary outcomes of this study– COVID-19-attributable hospitalizations and all-cause hospitalizations and deaths– were captured during the 28-day post-index acute period. A COVID-19-attributable hospitalization was defined as a hospital visit in which COVID-19 was listed in the primary diagnosis field during the acute period. All-cause hospitalizations were defined as any hospital visits that occurred during the acute period. Deaths reported in the acute period were also reported.

The secondary outcomes of this study described the proportion of patients treated during each of the six distinct 3- to 8-week periods of Omicron subvariants activity; the occurrence of COVID-19-attributable hospitalizations during the acute period was described for each of the six periods, and treatment outcomes were compared between period 1 and the other five periods.

Primary outcomes were also reported in two subpopulations of interest: severe renal disease or active cancer. Severe renal disease (based on ICD-10 diagnosis codes) included patients with stage 4 or 5 chronic kidney disease, those in receipt of peritoneal dialysis or hemodialysis, or those who had undergone kidney transplant (the latter group were not counted in the solid organ transplant recipients). Active cancer was defined as patients with cancer (based on a relevant cancer code at any time prior to assumed sotrovimab administration) who had received chemotherapy or radiotherapy within the 12 months prior to their index date.

### Data analysis

Continuous variables, such as age, were summarized using mean, standard deviation, median, interquartile range, and range. Categoric variables, such as sex, were described using frequencies and percentages. Small number suppression was applied for all small numbers up to, and including, seven by being rounded to the nearest five (regardless of the actual number). These values were replaced with an asterisk.

Incidence rates (per 100 patient-days) within 28 days were calculated as the number of hospitalizations divided by the total person time observed in days and amplified by 100. To compare incidence rates between period 1 (Omicron BA.1 predominance and BA.2 prevalence of less than 25%) and each of the other five periods, a multivariate Poisson regression model was used to estimate incidence rate ratios and associated confidence intervals for each period. The estimates were adjusted for patient age, previous COVID-19 admission, evidence of at least one high-risk comorbidity in the patient record, and time period of index.

## Results

### Patient demographics and baseline characteristics

In total, 10,096 patients were eligible for inclusion in the study (Table 2). The mean age of patients was 56.4 years and 42.0% of the study population was female (*n* = 4238). The percentage of patients who had a previous hospital admission in which COVID-19 was listed as a primary or underlying diagnosis was 3.0% (*n* = 298). The percentage of patients with evidence of at least one comorbidity was lowest (72.7%) in period 3 and highest (75.8%) in period 1, with no (descriptive) trend across the periods. Of the high-risk comorbidities, the most frequently reported were immune-mediated inflammatory disorders (IMIDs; 43.0%, *n* = 4337), severe renal disease (14.1%, *n* = 1422), rare neurologic conditions (10.4%, *n* = 1053), and active cancer (9.0%, *n* = 910). There was no evidence of high-risk comorbidities (based on available diagnosis codes) in 26.1% (*n* = 2633) of included patients.

**Table 2:** Patient characteristics

Characteristic	Patients (*n* = 10,096)
Age, years	
Mean (SD)	56.40 (16.4)
Median (IQR)	57 (44–69)
Age group, years, n (%)	
12–54	4412 (43.7)
55–64	2278 (22.6)
65–74	1961 (19.4)
≥ 75	1445 (14.3)
Female sex, n (%)	4238 (42)
Ethnicity, n (%)^a^	
White	6955 (68.9)
Asian/Asian British	619 (6.1)
Black/Black British/Caribbean or African	239 (2.4)
Mixed	114 (1.1)
Other	329 (3.3)
Unknown	1840 (18.2)
Previous admission for COVID-19, n (%)	298 (3.0)
High-risk comorbidities, n (%)^c^	
Active cancer	910 (9.0)
Down syndrome	107 (1.1)
HIV	^b^
Immune deficiencies^d^	338 (3.3)
Patients being treated for immune-mediated inflammatory disorders^e^	4337 (43.0)
Patients with hematologic diseases and stem cell transplant recipients	602 (6.0)
Patients with liver disease	438 (4.3)
Rare neurologic conditions^f^	1053 (10.4)
Severe renal disease	1422 (14.1)
Solid organ transplant recipients	280 (2.8)
No comorbidity, n (%)	2633 (26.1)

^a^ Percentages calculated based on removal of “unknown” group from the denominator;

^b^ Small number suppression applied

^c^ As defined by the UK Medicines & Healthcare products Regulatory Agency [13]

^d^ common variable immunodeficiency; undefined primary antibody deficiency on immunoglobulin (or eligible for IgG); hyper-IgM syndromes; Good’s syndrome; severe combined immunodeficiency; autoimmune polyglandular syndromes/autoimmune polyendocrinopathy, ectodermal dystrophy; primary immunodeficiency associated with impaired type I interferon signalling; X-linked agammaglobulinaemia (and other primary agammaglobulinaemias); any patient with a secondary immunodeficiency likely to be eligible for receipt of immunoglobulin replacement therapy

^e^ IMID treated with rituximab or other B cell depleting therapy in the last 12 months; IMID with active/unstable disease on corticosteroids, cyclophosphamide, tacrolimus, cyclosporin or mycophenolate; IMID with stable disease on either corticosteroids, cyclophosphamide, tacrolimus, cyclosporin or mycophenolate; IMID patients with active/unstable disease including those on biological monotherapy and on combination biologicals with thiopurine or methotrexate

^f^ multiple sclerosis; motor neurone disease; myasthenia gravis; Huntington’s disease

COVID-19, coronavirus disease 2019; HIV, human immunodeficiency virus; IMID, immune-mediated inflammatory disorders; IQR, interquartile range; SD, standard deviation

### Acute period outcomes

Acute period outcomes during the full study period (January 1–July 31, 2022) are presented in Table 3. COVID-19-attributable hospitalizations were recorded in 1.0% (*n* = 96) of patients; 72% of patients with a non-elective COVID admission event and 74% of those with no event had evidence of at least one comorbidity. Among the 96 patients with a COVID-19-attributable hospitalization, 49 had an IMID diagnosis (1.1% of all patients who had an IMID comorbidity). The percentage of patients who had a hospital visit due to any cause during the acute period following their sotrovimab treatment was 4.6% (*n* = 465). Overall, 0.3% (*n* = 27) of patients were recorded as having died due to any cause during the acute period.

**Table 3:** Overall acute period (28 days following index) outcomes

Outcome	Patients (*n* = 10,096)
COVID-19-attributable hospitalization, n (%)	96 (1)
Any-cause hospitalization, n (%)	465 (4.6)
All-cause deaths, n (%)	27 (0.3)

COVID-19, coronavirus disease 2019

#### Acute period outcomes for patients with advanced renal disease and active cancer

Among 1422 patients with severe renal disease, 1.3% (*n* = 18) had a COVID-19-attributable hospitalization during the acute period, 6.8% (*n* = 97) had a hospitalization due to any cause, and 0.3% (*n* = 4) died due to any cause (Table 4).

**Table 4:** Acute period outcomes (28 days following index): patients with severe renal disease and active cancer

Outcome	Severe renal disease (*n* = 1422)	Active cancer (*n* = 910)
COVID-19-attributable hospitalization, n (%)	18 (1.3)	10 (1.1)
Any-cause hospitalization, n (%)	97 (6.8)	89 (9.8)
All-cause deaths, n (%)	4 (0.3)	7 (0.8)

COVID-19, coronavirus disease 2019

Out of 910 patients who were identified as having an active cancer, 1.1% (*n* = 10) had a COVID-19-attributable hospitalization during the acute period, 9.8% (*n* = 89) had a hospitalization for any cause, and 0.8% (*n* = 7) died due to any cause (Table 4).

#### Acute period outcomes across periods of Omicron subvariants prevalence

Acute period outcomes according to the time of diagnosis in the six periods of Omicron subvariants prevalence are shown in Table 5. The proportions of patients with a COVID-19-attributable hospitalization across periods 1 to 6 were 1.0% (*n* = 22/2102), 1.3% (*n* = 13/993), 1.0% (*n* = 37/3884), 1.0% (*n* = 6/573), 1.4% (*n* = 16/1161), and 0.7% (*n* = 10/1383), respectively. This equated to an incidence rate per 100 patient-days of 0.040 for period 1, 0.050 for period 2, 0.036 for period 3, 0.040 for period 4, 0.052 for period 5, and 0.028 for period 6. A multivariate Poisson regression model found no evidence of significant differences in incidence of COVID-19-attributable hospitalizations for periods 2–6 (*p* values ranged from 0.13 to 0.83) relative to period 1 adjusted for age at diagnosis, previous admission for COVID-19, or evidence of at least one high-risk condition (Table 5).

**Table 5:** Acute period outcomes (28 days following index) across periods of Omicron subvariants prevalence

Outcome	Period 1 (BA.1 predominant, BA.2 < 25% prevalence) (*n* = 2102)	Period 2 (25% > BA.2 < 75% prevalence) (*n* = 993)	Period 3 (BA.2 > 75% prevalence) (*n* = 3884)	Period 4 (BA.5 < 25% prevalence) (*n* = 573)	Period 5 (25% > BA.5 < 75% prevalence) (*n* = 1161)	Period 6 (BA.5 > 75% prevalence) (*n* = 1383)
COVID-19-attributable hospitalization, n (%)	22 (1.0)	13 (1.3)	37 (1.0)	6 (1.0)	16 (1.4)	10 (0.7)
Incidence rate per 100 patient-days	0.040	0.050	0.036	0.040	0.052	0.028
Incidence rate ratio^a^ (95% CI)	REF	1.16 (0.58–2.31)	0.76 (0.44–1.30)	0.8 (0.32–1.99)	1.07 (0.56–2.06)	0.56 (0.26–1.19)
*P*-value	REF	0.67	0.31	0.63	0.83	0.13

^a^ Incidence of hospitalization = (hospitalizations observed/total person time in days) × 100

CI, confidence interval; COVID-19, coronavirus disease 2019; REF, reference group

## Discussion

We investigated patient characteristics and outcomes (COVID-19-attributable hospitalizations, all-cause hospitalizations, and deaths) in patients diagnosed with COVID-19 who received sotrovimab administered in NHS hospitals across England. The results of this study demonstrate that patients who were treated with sotrovimab in England between January 1 and July 31, 2022 experienced low levels of COVID-19-attributable hospitalizations during the 28 days following treatment administration. COVID-19-attributable hospitalizations were also low in subgroups of patients with severe kidney disease and active cancers. The most frequently reported high-risk comorbidity was an IMID (*n* = 4337; 43% of included patients); the therapeutic approach to COVID-19 in these patients is a subject of debate [15, 16]. In the present study, the observed rate of COVID-19-attributable hospitalization among patients with an IMID (1.1%) was similar to that reported for the overall population and other subgroups. Continuous low rates of clinical outcomes such as all-cause and COVID-19-attributable hospitalizations or deaths were also reported across subvariant predominance periods (BA.1, BA.2, and BA.5). Moreover, the analysis of COVID-19-attributable hospitalization rates between Omicron BA.1 (period 1), BA.2 (periods 2 and 3), and BA.5 activity (periods 4–6) indicated that there was no evidence of difference between period 1 and the other five periods.

The lack of pharmacy data in HES required indirect identification of assumed treatment with sotrovimab, which is a potential limitation of our study. However, we consider HES to be the most appropriate source of data for our study, as it provides comprehensive details of hospital care in England [17]. In addition, the patient selection algorithm was based on commissioning guidelines for use of sotrovimab, which should increase confidence in the indirect identification. Moreover, 99.98% of non-hospitalized patients who were treated with a mAb during the study period received sotrovimab [10]. Our results are consistent with those from a study, conducted between December 16, 2021, and February 10, 2022, using the OpenSAFELY-TPP platform, which reported that 0.96% of patients confirmed to have been treated with sotrovimab had a COVID-19-attributable hospitalization or death within 28 days of treatment [18]; in our study, 1.0% of patients who were assumed to have been treated with sotrovimab experienced a COVID-19-attributable hospitalization in the 28-day post-treatment acute period. The results are also similar to those of another recently completed analysis that used data from the Discover database in Northwest London, which reported 0.7% of patients confirmed to have been treated with sotrovimab experiencing a COVID-19-attributable hospitalization during the 28 days following treatment (study period was December 1, 2021–May 31, 2022, with subvariant predominance as follows: Omicron BA.1 from December 1, 2021–February 28, 2022, and Omicron BA.2 from March 1–May 31, 2022) [19]. Similarly, our findings of low rates of COVID-19-attributable deaths and hospitalizations in patients with advanced kidney disease are consistent with those from a recent study in non-hospitalized patients with COVID-19 on kidney replacement therapy; treatment with sotrovimab resulted in a substantially lower risk of severe COVID-19 outcomes compared with molnupiravir during periods of Omicron BA.1 through to BA.5 subvariant dominance [20]. Finally, our findings are also consistent with the most recent data from the OpenSAFELY-TPP platform, comparing the effectiveness of sotrovimab and nirmatrelvir/ritonavir in preventing severe COVID-19 outcomes when different subvariants of Omicron were dominant [21]; the risk of severe outcomes was similar between the treatment groups, with no changes observed due to circulation of the BA.5 subvariant.

Recent studies have demonstrated that the Omicron BA.2 variant is similar in severity to the Omicron BA.1 variant [14, 18, 22, 23], although it may have increased severity in certain populations such as the elderly [24]. Our large, population-based study across England contributes to the overall favorable weight of evidence to support the clinical benefit of sotrovimab as an early treatment for COVID-19 through Omicron subvariant predominance periods, especially for patients at higher risk of developing severe symptoms, such as those with severe renal diseases and active cancer. Moreover, our findings also confirm those of a recent study that reported similar proportions of hospital admissions between sequence-confirmed Omicron BA.1 and BA.2 cases treated with sotrovimab [25]. In addition, our study further extends these findings by also assessing patients treated during periods of Omicron BA.5 prevalence.

These data, in conjunction with preclinical data supporting in vitro and in vivo antiviral activity of sotrovimab against Omicron BA.2 and Omicron BA.5 variants, reinforce the lack of validated models to predict correlates of efficacy based solely on in vitro neutralization [26, 27]. The variability of in vitro results, based on cell culture and assay systems and a lack of models to incorporate the role of Fc effector function (which triggers the body’s own innate immune cells to fight SARS-CoV-2 infection, thus contributing to sotrovimab effectiveness), may also compromise the ability to reproduce clinical effects in vitro. Further investigation into the relationship between in vitro and in vivo antiviral activity would be valuable, for example through additional preclinical models or randomized controlled trials (although the latter are difficult to conduct in the context of a constantly evolving variant landscape). In lieu of further studies and evidence, the totality of available evidence including in vitro, in vivo, and observational data should be considered when determining treatment options for early SARS-CoV-2 infection.

We used an adjusted multivariate Poisson regression model to estimate incidence rate ratios, although we cannot exclude the possibility that unmeasured confounders (such as severity of symptoms) impacted our findings. However, since the eligibility criteria for receiving sotrovimab were similar in the periods of BA.1 and BA.2 variant predominance, there is no reason to expect a difference in symptom severity between the periods.

Another important limitation of this study is the single-arm design, which prevented any comparison with a reference group of patients. Administration of oral anti-virals is not captured within HES due to the lack of pharmacy data; therefore, comparison with these agents (or confirmation of untreated groups) was not feasible with this data source. The absence of accurate data for SARS-CoV-2 positive infections in the community also contributed to the absence of an untreated comparator group. In addition, comorbidities are known to be under-reported in the HES database [28], so adequately controlling for all potential confounders would be difficult.

The known under-reporting of comorbidities within the HES database means that the characterization of high-risk comorbidities amongst sotrovimab-treated patients may be incomplete. In the current study, 26% of the total population did not have any comorbidities recorded. Moreover, recording only the comorbidities that have been noted as part of hospital in- or outpatient activity will also introduce bias towards identification of clinically impactful and active comorbidities. Furthermore, the classification of each patient as a high-risk case relies on the associated diagnoses being recorded with an admission event for the identified patients. This may result in underestimation of some high-risk conditions, further compounded by the lack of pharmacy data on prescribed medicines. Also, as a given comorbidity has to have been severe enough to warrant review in hospital, and as many regular reviews of chronic conditions were likely deprioritized during the pandemic, this may have contributed to under-reporting. As a high-cost drug, sotrovimab is unlikely to be approved for patients without a diagnosis fitting the eligibility criteria in the latest guidance [13]. Within this cohort, the variable, as described, could then more strictly be interpreted as acting as a proxy for those patients requiring recent hospital care where their diagnosis is noted.

Confirmatory polymerase chain reaction test results for COVID-19 were not available for the patients included in this study. We therefore used sequence analysis data published by the UK Health Security Agency as a proxy for the Omicron subvariants most likely to be in circulation during the different study periods; as a result, there is a risk of ecological bias. However, initial studies suggest clinical coding of COVID-19 in HES is of good quality (England 2021), and an expanded COVID-19 clinical coding policy had been in place for over a year at the time of the study period, so impacts on our findings due to uncertainty around subvariant classification is expected to be minimal [29, 30]. It is not possible to consistently distinguish planned and unplanned single overnight stays in HES data; therefore, in order to restrict included patients to the directed use of sotrovimab, all overnight stays were excluded. This may exclude some patients who were effectively hospitalized on Day 0 following their treatment, although they would have to deteriorate substantially immediately after receiving their sotrovimab treatment and would probably not be eligible to receive sotrovimab in the NHS in England [6]. Also, COVID-19 vaccination status, which is likely linked to the probability of subsequently being admitted due to COVID-19, was not available in the study dataset. However, vaccination rates in the study population are expected to be higher than in the general population due to their higher risk for poor COVID-19 outcomes and a longer time in which the vaccine was available to them. Lastly, since the study aimed to assess acute clinical outcomes (up to 28 days from treatment administration), we are unable to report data for outcomes beyond this time period, including COVID-19 relapse or SARS-CoV-2 reinfection.

## Conclusion

Patients assumed to have been treated with sotrovimab experienced low levels of COVID-19-attributable hospitalizations and all-cause deaths across periods of different Omicron subvariant prevalence. The results were consistent within subgroups of patients with severe renal disease and active cancer, as well as across periods of Omicron BA.1, BA.2, and BA.5 subvariant activity. No evidence of differences in hospitalization rates were observed during different periods aligned with prevalence of Omicron BA.1 and periods of BA.2 or BA.5 subvariant predominance.

## Data Availability

The datasets generated and/or analyzed during the current study are available in NHS Digital via the Data Access Request Service (DARS) repository. Copyright © 2023, re-used with the permission of NHS Digital.

## Declarations

Harvey Walsh Ltd (Part of the OPEN Health group) are licensed by NHS Digital to receive Hospital Episode Statistics data under data-sharing agreement DARS-NIC-05934-M7V9K and follow NHS Digital guidelines. The IGARD advisory body to NHS Digital gave approval for the use of HES data for purposes including, but not limited to, epidemiology research and health economic research.

## Abbreviations

COMET-ICE: COVID-19 Monoclonal antibody Efficacy Trial-Intent to Care Early
COVID-19: Coronavirus disease 2019
HES: Hospital Episode Statistics
ICD-10: International Statistical Classification of Diseases and Related Health Problems 10th revision
mAb: Monoclonal antibody
NHS: National Health Service
SARS-CoV-2: Severe acute respiratory syndrome coronavirus 2

## References

[1] Gaffney AW, Himmelstein DU, Woolhandler S (2020). Illness-related work absence in Mid-april was highest on record. JAMA Intern Med.

[2] Gebru AA, Birhanu T, Wendimu E, Ayalew AF, Mulat S, Abasimel HZ (2021). Global burden of COVID-19: situational analyis and review. Hum Antibodies.

[3] Cathcart AL, Havenar-Daughton C, Lempp FA, Ma D, Schmid M, Agostini ML et al. The dual function monoclonal antibodies VIR-7831 and VIR-7832 demonstrate potent in vitro and in vivo activity against SARS-CoV-2. bioRxiv. 2022; 10.1101/2021.03.09.434607

[4] Gupta A, Gonzalez-Rojas Y, Juarez E, Crespo Casal M, Moya J, Rodrigues Falci D (2022). Effect of sotrovimab on hospitalization or death among high-risk patients with mild to moderate COVID-19: a randomized clinical trial. JAMA.

[5] European Medicines Agency. Xevudy (sotrovimab) authorization. https://www.ema.europa.eu/en/medicines/human/EPAR/xevudy. Accessed 7 March 2024.

[6] Medicines & Healthcare products Regulatory Agency. Summary of product characteristics for Xevudy. https://www.gov.uk/government/publications/regulatory-approval-of-xevudy-sotrovimab/summary-of-product-characteristics-for-xevudy. Accessed 7 February 2024.

[7] Medicines & Healthcare products Regulatory Agency. Last updated 19/10/22 - summary of product characteristics for Lagevrio. https://www.gov.uk/government/publications/regulatory-approval-of-lagevrio-molnupiravir/summary-of-product-characteristics-for-lagevrio. Accessed 7 February 2024.

[8] NHS England. Interim Clinical Commissioning Policy: treatments for hospital-onset COVID-19. https://www.england.nhs.uk/coronavirus/documents/interim-clinical-commissioning-policy-antivirals-or-neutralising-monoclonal-antibodies-in-the-treatment-of-hospital-onset-covid-19/. Accessed 17 Aug 2023.

[9] Park YJ, Pinto D, Walls AC, Liu Z, De Marco A, Benigni F (2022). Imprinted antibody responses against SARS-CoV-2 Omicron sublineages. Science.

[10] NHS England. COVID-19 therapeutics: antivirals and neutralising-monoclonal antibodies (AVs and nMABs): non-hospitalised treatments - national summary. Accessed 7 February 2024. https://view.officeapps.live.com/op/view.aspx?src=https%3A//www.england.nhs.uk/statistics/wp-content/uploads/sites/2/2023/01/COVID-THERAPEUTICS-WEEKLY-PUBLICATION-week-ending-080123-V2.0-OCT-22-Onwards.xlsx&wdOrigin=BROWSELINK

[11] World Health Organization. International Statistical Classification of Diseases and Related Health Problems 10th Revision (ICD-10) WHO version. https://icd.who.int/browse10/2019/en#/U07.2. Accessed 7 February 2024.

[12] NHS Digital. National Clinical Coding Standards OPCS-4. (2006). https://nhsengland.kahootz.com/t_c_home/view?objectId=15799120. Accessed 7 February 2024.

[13] Medicines & Healthcare products Regulatory Agency. Central Alerting System. Treatments for highest risk non-hospitalised patients (adults and children) with COVID-19. https://www.cas.mhra.gov.uk/ViewandAcknowledgment/ViewAlert.aspx?AlertID=103218. Accessed 7 February 2024.

[14] UK Health Security Agency. SARS-CoV-2 variants of concern and variants under investigation in England. Technical briefing 45. https://assets.publishing.service.gov.uk/government/uploads/system/uploads/attachment_data/file/1115071/Technical-Briefing-45-9September2022.pdf. Accessed 7 February 2024.

[15] Lahouati M, Cazanave C, Labadie A, Gohier P, Guirlé L, Desclaux A (2023). Outcomes of targeted treatment in immunocompromised patients with asymptomatic or mild COVID-19: a retrospective study. Sci Rep.

[16] Orth HM, Flasshove C, Berger M, Hattenhauer T, Biederbick KD, Mispelbaum R et al. Early combination therapy of COVID-19 in high-risk patients. Infection. 2023; Nov 29. 10.1007/s15010-023-02125-5 10.1007/s15010-023-02125-5 PMC11142969 38017344

[17] NHS England. The processing cycle and HES data quality. https://digital.nhs.uk/data-and-information/data-tools-and-services/data-services/hospital-episode-statistics/the-processing-cycle-and-hes-data-quality. Accessed 7 February 2024.

[18] Zheng B, Green ACA, Tazare J, Curtis HJ, Fisher L, Nab L (2022). Comparative effectiveness of sotrovimab and molnupiravir for prevention of severe covid-19 outcomes in patients in the community: observational cohort study with the OpenSAFELY platform. BMJ.

[19] Patel V, Yarwood MJ, Levick B, Gibbons DC, Drysdale M, Kerr W (2022). Characteristics and outcomes of patients with COVID-19 at high-risk of disease progression receiving sotrovimab, oral antivirals or no treatment in England. medRxiv.

[20] Zheng B, Campbell J, Carr EJ, Tazare J, Nab L, The OpenSAFELY Collaborative (2022). Comparative effectiveness of sotrovimab and molnupiravir for preventing severe COVID-19 outcomes in non-hospitalised patients on kidney replacement therapy: observational cohort study using the OpenSAFELY-UKRR linked platform and SRR database. medRxiv.

[21] Zheng B, Tazare J, Nab L, Mehrkar A, MacKenna B, Goldacre B (2023). Comparative effectiveness of Paxlovid versus sotrovimab and molnupiravir for preventing severe COVID-19 outcomes in non-hospitalised patients: observational cohort study using the OpenSAFELY platform. medRxiv.

[22] Lewnard JA, Hong VX, Patel MM, Kahn R, Lipsitch M, Tartof SY (2022). Clinical outcomes associated with SARS-CoV-2 Omicron (B.1.1.529) variant and BA.1/BA.1.1 or BA.2 subvariant infection in Southern California. Nat Med.

[23] Wolter N, Jassat W, DATCOV-Gen author group, von Gottberg A, Cohen C. Clinical severity of omicron lineage BA.2 infection compared with BA.1 infection in South Africa. Lancet. 2022;400:93–6. 10.1016/S0140-6736(22)00981-3 PMC9246473 35780802

[24] Gautret P, Hoang VT, Jimeno MT, Lagier JC, Rossi P, Fournier PE (2022). The severity of the first 207 infections with the SARS-CoV-2 Omicron BA.2 variant, in Marseille, France, December 2021-February 2022. J Med Virol.

[25] Harman K, Nash SG, Webster HH, Groves N, Hardstaff J, Bridgen J (2023). Comparison of the risk of hospitalisation among BA.1 and BA.2 COVID-19 cases treated with sotrovimab in the community in England. Influenza Other Respir Viruses.

[26] Bruel T, Hadjadj J, Maes P, Planas D, Seve A, Staropoli I (2022). Serum neutralization of SARS-CoV-2 Omicron sublineages BA.1 and BA.2 in patients receiving monoclonal antibodies. Nat Med.

[27] Case JB, Mackin S, Errico JM, Chong Z, Madden EA, Whitener B (2022). Resilience of S309 and AZD7442 monoclonal antibody treatments against infection by SARS-CoV-2 Omicron lineage strains. Nat Commun.

[28] Navid A, Hajibandeh S, Mohan J, Hajibandeh S (2015). Improving the accuracy of HES comorbidity codes by better documentation in surgical admission proforma. Br J Hosp Med (Lond).

[29] NHS. Clinical practice guide for improving the management of adult COVID-19 patients in secondary care. https://yorksandhumberdeanery.nhs.uk/sites/default/files/covid19_clinical_practice_guidance_g2.pdf. Accessed 7 February 2024.

[30] NHS England. Appendix 2 - Multidisciplinary Coded Dataset Suggestions (MCDS) to improve accuracy and consistency of COVID-19 coding. https://www.gettingitrightfirsttime.co.uk/wp-content/uploads/2018/02/COVID19-MCDS-Appendix-Feb21-FINAL.pdf. Accessed 7 February 2024.

